# Global Research Trends in Pediatric COVID-19: A Bibliometric Analysis

**DOI:** 10.3389/fpubh.2022.798005

**Published:** 2022-02-16

**Authors:** Siyu Hu, Xi Wang, Yucong Ma, Hang Cheng

**Affiliations:** ^1^Department of Pediatrics, The First Hospital of Jilin University, Changchun, China; ^2^Department of Clinical Laboratory, The First Hospital of Jilin University, Changchun, China

**Keywords:** COVID-19, childhood, research hotspot, bibliometric analysis, VOSviewer, research trends

## Abstract

**Background:**

Coronavirus disease 2019 (COVID-19) emerged in 2019 and has since caused a global pandemic. Since its emergence, COVID-19 has hugely impacted healthcare, including pediatrics. This study aimed to explore the current status and hotspots of pediatric COVID-19 research using bibliometric analysis.

**Methods:**

The Institute for Scientific Information Web of Science core collection database was searched for articles on pediatric COVID-19 to identify original articles that met the criteria. The retrieval period ranged from the creation of the database to September 20, 2021. A total of 3,561 original articles written in English were selected to obtain data, such as author names, titles, source publications, number of citations, author affiliations, and countries where the studies were conducted. Microsoft Excel (Microsoft, Redmond, WA) was used to create charts related to countries, authors, and institutions. VOSviewer (Center for Science and Technology Studies, Leiden, The Netherlands) was used to create visual network diagrams of keyword, author, and country co-occurrence.

**Results:**

We screened 3,561 publications with a total citation frequency of 30,528. The United States had the most published articles (1188 articles) and contributed the most with author co-occurrences. The author with the most published articles was Villani from the University of Padua, Italy. He also contributed the most co-authored articles. The most productive institution was Huazhong University of Science and Technology in China. The institution with the most frequently cited published articles was Shanghai Jiao Tong University in China. The United States cooperated most with other countries. Research hotspots were divided into two clusters: social research and clinical research. Besides COVID-19 and children, the most frequent keywords were pandemic (251 times), mental health (187 times), health (172 times), impact (148 times), and multisystem inflammatory syndrome in children (MIS-C) (144 times).

**Conclusion:**

Pediatric COVID-19 has attracted considerable attention worldwide, leading to a considerable number of articles published over the past 2 years. The United States, China, and Italy have leading roles in pediatric COVID-19 research. The new research hotspot is gradually shifting from COVID-19 and its related clinical studies to studies of its psychological and social impacts on children.

## Introduction

There is an ongoing worldwide pandemic involving a novel virus belonging to the family of coronaviruses (CoVs), including large, enveloped, positive-sense single-stranded RNA viruses (1). The virus that caused this pandemic is the severe acute respiratory syndrome coronavirus 2 (SARS-CoV-2), and the disease it causes is the coronavirus disease 2019 (COVID-19). As of September 20, 2021, 228,506,698 COVID-19 cases have been confirmed, including 4,692,361 deaths reported to the World Health Organization (2), and this number continues to increase.

According to current epidemiological trends, children are less likely to be infected with SARS-CoV-2 than adults, and most pediatric cases are asymptomatic or mild (3). Pediatric COVID-19 typically presents with mild symptoms, such as cough, fever, sore throat, and diarrhea. However, some children, especially those with underlying diseases, may present with severe clinical manifestations such as hyperinflammatory syndrome (4, 5), increased severity of childhood-onset type 1 diabetes (6), and multisystem inflammatory syndrome (7). Acute myocarditis with intense systemic inflammation and atypical Kawasaki disease have also emerged as severe pediatric diseases after SARS-CoV-2 infection (4, 8).

Because most children with COVID-19 are asymptomatic or have mild symptoms, supportive treatment is usually sufficient, and hospitalization is unnecessary (9). Ensuring sufficient calorie and water intake, maintaining water-electrolyte balance and homeostasis, and strengthening psychotherapy for older children usually comprise adequate treatment (10). COVID-19 vaccines have been determined to be safe for adults, and trials to determine their safety for children are ongoing. When the evidence or epidemiological situation justifies a change in the vaccine policy, the World Health Organization will update its recommendations (11). With the continuing COVID-19 pandemic, children's mental health has begun to receive more attention. Many factors such as fear influence children's mental health. COVID-19 has instilled fear in children because they are worried about becoming sick and their parents having to stay home from work (12). Furthermore, changes in family economic conditions, protective confinement, social isolation, and deaths of loved ones have had varying degrees of effect on children's mental health (13–15). Therefore, innovative approaches that increase access to mental health services and promote resilience and mental well-being, such as maintaining social connections despite the isolation and renewing social ties during the recovery phase, should be explored. Similarly, increasing the identification of and support for children, adolescents, and families experiencing disproportionate effects of the pandemic and implementing preventive measures may reduce long-term mental health sequelae for children and adolescents (16).

Bibliometrics, the method we use in this study, applies statistics and mathematics to analyze written publications such as books and journal articles. Bibliometric methods can be used to analyze scientific literature in a specific field to assess the popularity and impact of specific publications, authors, and institutions. Bibliometric analyses provide quantitative and qualitative evaluations of publications and provide the developing trends of research domains (17). So far, there was one article about bibliometric analyses of pediatric COVID-19 that studied the articles for 6 months, from January 1, 2020, to June 11, 2020 (18). There were no similar bibliometric analyses of pediatric COVID-19 after June 11, 2020. Thus, in this study, we performed a bibliometric analysis to analyze various aspects of pediatric COVID-19 from the COVID-19 outbreak to September 20, 2021 and further identified its research hotspots and trends.

## Materials and Methods

### Data Sources and Search Strategies

To perform our search, we used the Web of Science database, which is often used for bibliometric analyses because of its strict assessment of publications and high-quality literature (19). To avoid bias caused by database updates, all data were retrieved and exported on September 20, 2021. The search strategy used the following keywords: [AK=(child^*^ OR baby^*^ OR newborn^*^ OR toddler ^*^ OR infant OR pediatric^*^) OR TI=(child^*^ OR baby^*^ OR newborn^*^ OR toddler^*^ OR infant OR pediatric^*^)] AND [AK=(“COVID 19” OR “coronavirus 2019” OR “coronavirus disease 2019” OR SARS-CoV-2 OR 2019-nCoV OR “2019 novel coronavirus” OR “SARS coronavirus 2” OR “Severe Acute Respiratory Syndrome Coronavirus-2” OR SARS-COV2) OR TI=(“COVID 19” OR “coronavirus 2019” OR “coronavirus disease 2019” OR SARS-CoV-2 OR 2019-nCoV OR “2019 novel coronavirus” OR “SARS coronavirus 2” OR “Severe Acute Respiratory Syndrome Coronavirus-2” OR SARS-COV2)]. The inclusion criteria were publications written in English and original articles. The exclusion criteria were non-English articles and publications other than original articles.

### Data Extraction

Two independent researchers extracted relevant data from screened articles, including titles, keywords, authors, institutions, journals, countries and regions, and total citations.

### Bibliometric Analysis

Microsoft Excel 2016 (Microsoft, Redmond, WA) was used to perform statistical analyses of countries, institutions, articles, source publications, and authors. VOSviewer 1.6.16 (Center for Science and Technology Studies, Leiden, the Netherlands) was used to visualize keyword, author, and country co-occurrences. During network visualization, items were represented by their labels and, by default, by a circle. The sizes of the label and circle of an item were determined by their weight. The higher the weight of an item, the larger the label and circle for that item. To avoid overlapping labels, the labels may not be displayed for some items. The appearance of two items on the same line indicates that they co-occurred; the closer the two items, the more times they co-occurred. The same applied to the co-occurrences of countries, institutions, and authors. Additionally, we searched impact factors and quartiles of categories using Journal Citation Reports (JCR) 2020, which cites the data of more than 8,000 journals (online version), including 3,800 core journals (CD-ROM version) included in the Science Citation Index, and defines indexes such as the impact factor for each journal.

### Statistical Analyses

The appearing months are expressed as the mean ± standard deviation (SD). The data analyses were performed using GraphPad Prism software (version 8.0; GraphPad, Inc., La Jolla, CA, USA). Statistical significance was set at *P* < 0.05.

## Results

### Descriptions and Trends of Publications

**Figure 1:**
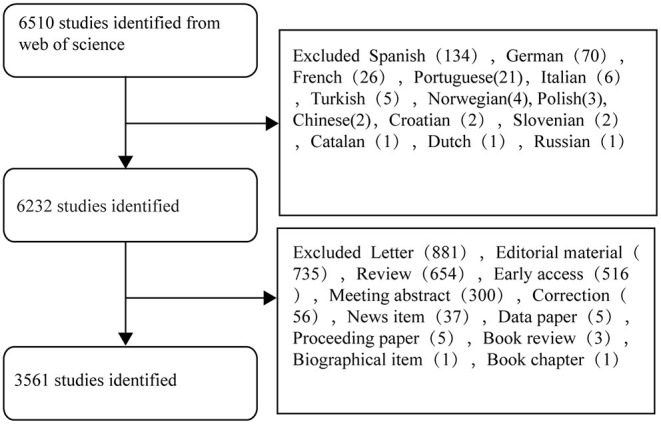
Flow diagram of the screening process used to identify publications about pediatric COVID-19.

A total of 6,510 publications on COVID-19 and children were retrieved from the Core Collection Database of Web of Science. We excluded non-English studies, early access, new items, reviews, and books. The flow diagram of the screening process is shown in Figure 1. Finally, 3,561 publications were included; the total citation frequency was 30,528. 1139 journals from 138 countries contributed to these publications. The first article on COVID-19 in children was published in February 2020 in China. Subsequently, more articles have been published, with 1357 articles published in 2020. The number of publications has dramatically increased, with 2204 available on September 20, 2021 (Supplementary Table 1).

### Ten Countries With the Most Articles

**Figure 2:**
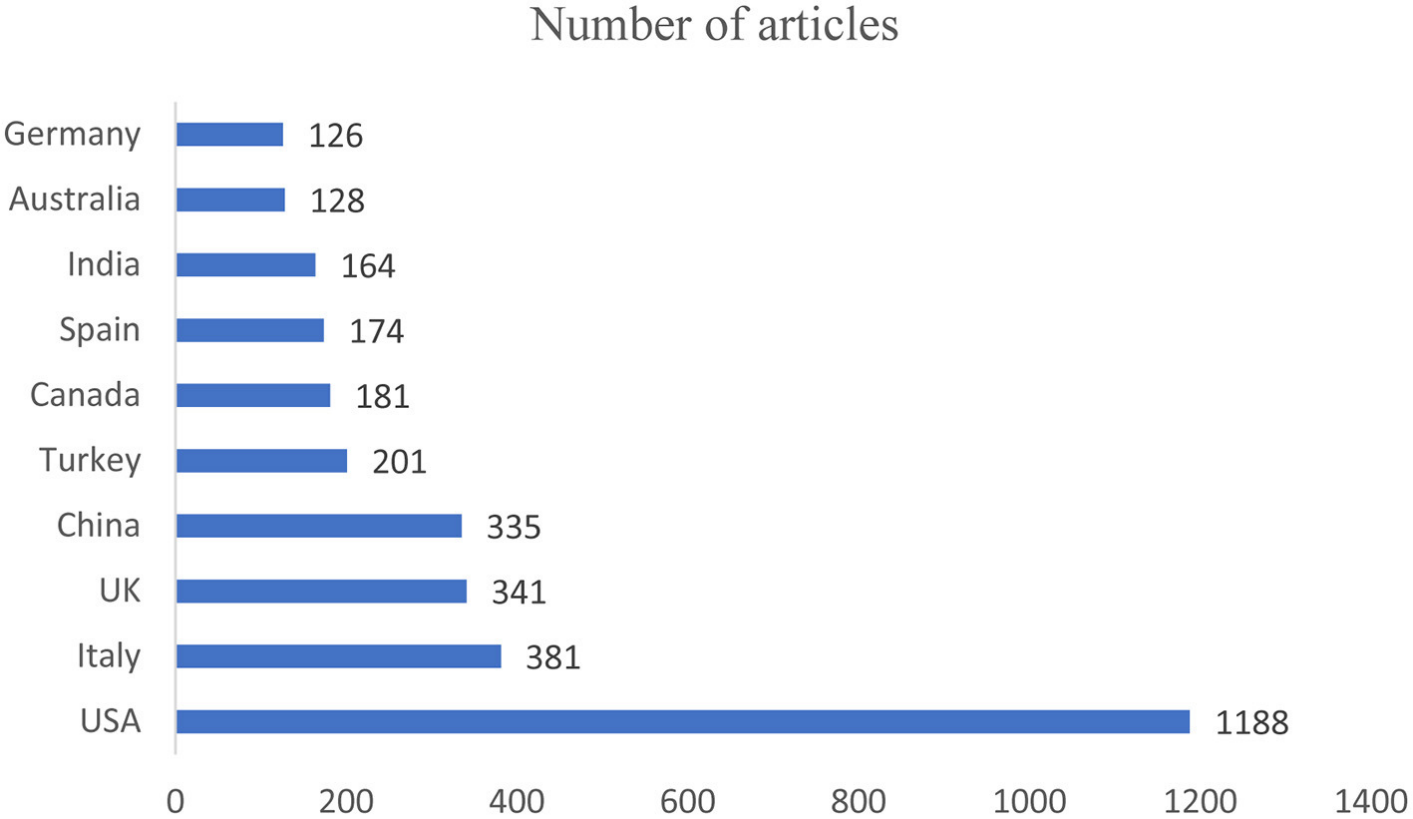
Ten countries with the most articles. The x-axis shows the numbers of articles, and the y-axis shows the names of the countries.

The total number of articles published by the 10 countries contributing the most articles was 3219, accounting for 90.35% of all eligible articles. The United States had 1188 articles, which was the largest number of articles, accounting for 33.34% of the total publications, followed by Italy with 381 articles, the United Kingdom with 341 articles, and China with 335 articles (Figure 2). Among the 10 countries with the most articles, five were in Europe.

### Ten Authors With the Greatest Number of Publications

The details of the 10 authors with the most published articles are shown in Table 1. These authors each had 11–18 articles published. Four authors were from Italy, three from the United States, and three from China. Villani, from the University of Padua in Italy, published the most articles (18). Shao, from Southern Medical University in China, published the second-highest number of articles (15) (Table 1).

**Table 1:** Ten authors with the greatest number of publications.

**Rank**	**Author**	**Number of articles**	**Affiliation**	**Country**
1	Villani A	18	University of Padua	Italy
2	Shao Jianbo	15	Huazhong University of Science and Technology	China
3	Buonsenso D	14	IRCCS Policlinico Gemelli	Italy
4a	Li Hui	11	Huazhong University of Science and Technology	China
4b	Bassiri H	11	University of Pennsylvania	USA
4c	Behrens EM	11	University of Pennsylvania	USA
4d	Campana A	11	IRCCS Bambino Gesu	Italy
4e	Chiotos K	11	Childrens Hospital of Philadelphia	USA
4f	Lanari M	11	University of Bologna	Italy
4g	Lu Xiaoxiao	11	Huazhong University of Science and Technology	China

### Ten Most Highly Cited Articles

The 10 most highly cited articles were filtered according to their number of citations, ranging from 314 to 1,586 (Table 2). These were from the United States and the United Kingdom. According to the Journal of Citation Reports 2020, nine were published in Q1, and one was in Q2. The journal categories included pediatrics, public, environmental, and occupational health, biochemistry and molecular biology, infectious diseases, general and internal medicine, cardiovascular system, and cardiology. The impact factor of the source journals ranged from 3.309 to 56.272. The most cited article was “Epidemiology of COVID-19 Among Children in China” by Dong, which was published in *Pediatrics*; its objective was to identify epidemiological characteristics and transmission patterns of pediatric patients with COVID-19 in China (20). Six of the 10 most cited articles were about clinical and epidemiological characteristics of pediatric COVID-19, and two were about the current situation encountered by children with other diseases in the context of the new coronary pneumonia epidemic. One was about differences between pediatric COVID-19 and adult COVID-19 cases and summarized common human viruses from recent years. The other is a modeling study about the indirect effects of the COVID-19 pandemic on maternal and child mortality in low-income and middle-income countries (Table 2).

**Table 2:** Ten most highly cited articles.

**Authors**	**Article title**	**Citation count**	**Quartile in category**	**Impact factor**	**Journal**	**Country**
Dong YY	Epidemiology of COVID-19 among children in China	1586	Q1	7.124	Pediatrics	USA
Bialek S	Coronavirus disease 2019 in children-United States, February 12-April 2, 2020	678	Q1	17.586	MMWR-Morbidity and mortality weekly report	USA
Xu Y	Characteristics of pediatric SARS-CoV-2 infection and potential evidence for persistent fecal viral shedding	650	Q1	53.44	Nature medicine	USA
Qiu HY	Clinical and epidemiological features of 36 children with coronavirus disease 2019 (COVID-19) in Zhejiang, China: an observational cohort study	529	Q1	25.071	Lancet infectious diseases	UK
Whittaker E	Clinical characteristics of 58 children with a pediatric inflammatory multisystem syndrome temporally associated with SARS-CoV-2	525	Q1	56.272	JAMA-Journal of the american medical association	USA
Xia W	Clinical and CT features in pediatric patients with COVID-19 infection: different points from adults	456	Q2	3.309	Pediatric pulmonology	USA
Toubiana J	Kawasaki-like multisystem inflammatory syndrome in children during the COVID-19 pandemic in Paris, France: prospective observational study	380	Q1	39.89	BMJ-British medical journal	UK
Belhadjer Z	Acute heart failure in multisystem inflammatory syndrome in children in the context of global SARS-CoV-2 pandemic	372	Q1	29.69	Circulation	UK
Roberton T	Early estimates of the indirect effects of the COVID-19 pandemic on maternal and child mortality in low-income and middle-income countries: a modeling study	319	Q1	26.763	Lancet global health	USA
Shekerdemian LS	Characteristics and outcomes of children with coronavirus disease 2019 (COVID-19) infection admitted to US and canadian pediatric intensive care units	314	Q1	16.193	JAMA pediatrics	USA

### Ten Institutions With the Most Articles and the Most Frequently Cited Articles

Among the 10 institutions with the most articles, seven were in the United States, one was in China, one was in Italy, and one was in the United Kingdom. Huazhong University of Science and Technology (76 articles) in China contributed the most articles, followed by Harvard Medical School in the United States, with 67 articles (Table 3). When the institutions with the most frequently cited articles were ranked from one to ten according to that frequency, Shanghai Jiao Tong University in China was number one, with 16 articles, followed by Huazhong University of Science and Technology and the University of Pennsylvania (Table 4).

**Table 3:** Ten institutions with the most articles.

**Rank**	**Institution**	**Number of articles**	**Citation**	**Average citation**	**Country**
1	Huazhong University of Science and Technology	76	1,910	25.1316	China
2	Harvard Medical School	67	877	13.0896	USA
3	University of Pennsylvania	62	1,837	29.629	USA
4	Boston Children's Hospital	61	541	8.8689	USA
5	Columbia University	57	1,116	19.5789	USA
6	Children's Hospital of Philadelphia	56	1,509	26.9464	USA
7	University of Milan	53	406	7.6604	Italy
8	University of Toronto	52	577	11.0962	UK
9	University of Washington	46	588	12.7826	USA
10	University of Colorado	43	227	5.2791	USA

**Table 4:** Ten institutions with the most frequently cited articles.

**Rank**	**Institution**	**Number of articles**	**Citation**	**Average citation**	**Country**
1	Shanghai Jiao Tong University	16	1,933	120.8125	China
2	Huazhong University of Science and Technology	76	1,910	25.1316	China
3	University of Pennsylvania	62	1,837	29.629	USA
4	Xi'an Jiao Tong University	6	1,623	270.5	China
5	Nanjing Medical University	8	1,607	200.875	China
6	Anhui Medical University	3	1,587	529	China
7	Children's Hospital of Philadelphia	56	1,509	26.9464	USA
8	Center for Disease Control and Prevention	19	1,395	73.4211	USA
9	Columbia University	57	1,116	19.5789	USA
10	University of Paris	24	1,033	43.0417	France

### Ten Journals With the Greatest Number of Published Articles

The 10 journals with the greatest number of published articles are shown in Table 5, with details including the number of articles, impact factor, total citations and country of origin. *Frontiers in Pediatrics* was the top journal with 106 published articles. The journal's impact factor is 3.418, and there were 429 citations. *The Pediatric Infectious Disease Journal* was the second most active journal, followed by *International Journal of Environmental Research and Public Health, Pediatrics, Cureus*, and *Pediatric Pulmonology*. *Pediatrics* has the highest impact factor with 7.125, and had the most citations. *The Pediatric Infectious Disease Journal* had the second highest number of citations.

**Table 5:** Ten active journals with the greatest number of articles.

**Rank**	**Source publication**	**Number of articles**	**Impact factor**	**Quartile in category**	**Citation**	**Country**
1	Frontiers in pediatrics	106	3.418	Q1	429	Switzerland
2	Pediatric infectious disease journal	85	2.219	Q4	1,061	USA
3	International journal of environmental research and public health	65	3.39	Q1	217	Switzerland
4	Pediatrics	64	7.125	Q1	2646	USA
5	Cureus	45	—	Q3	99	USA
6	Pediatric pulmonology	43	3.039	Q2	735	USA
7	European journal of pediatrics	42	3.183	Q1	207	Germany
8	Frontiers in psychology	40	2.988	Q2	5	Switzerland
9	Journal of the pediatric infectious diseases society	40	3.164	Q1	483	UK
10	Child abuse and neglect	37	3.928	Q1	204	USA

### Cluster Analysis of the Co-occurrences of Countries and Authors

**Figure 3:**
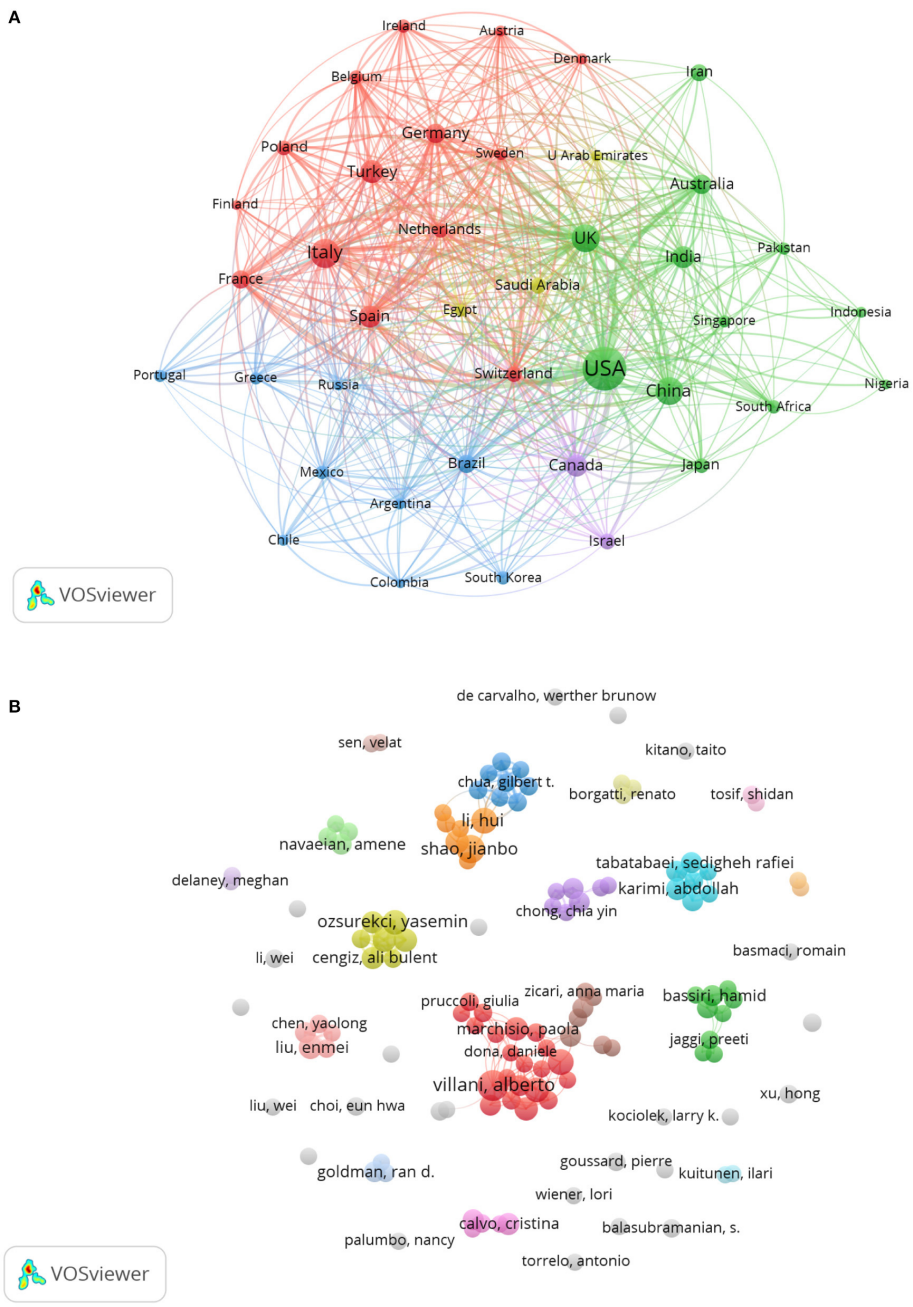
Analysis of the co-occurrence of countries and authors associated with publications on pediatric COVID-19. **(A)** Distribution of co-authorship among countries that formed clusters, with the same color representing one cluster. The number of co-authored articles contributed by each country is represented by the size of the circle: the higher contribution of co-authored articles, the larger the circle for the country. Cooperation among countries is represented by the distance between two countries: the closer the distance, the more frequent the cooperation among countries. **(B)** Distribution of co-authorship among authors. The larger the circle of an author, the more co-authored articles that author has contributed, and the more densely connected the lines between authors, the more collaborations they have.

We used VOSviewer (Center for Science and Technology Studies) to conduct a cluster analysis of the co-occurrence of countries. After setting the minimum number of documents of a country to 20, we found 40 countries (Figure 3A). After exporting the data related to the graph's content to Excel (Microsoft) and combining the analysis (Supplementary Table 2). By the order of link strength, USA, UK, Italy, Spain, Germany, France, Australia, Switzerland, Canada and Netherlands were the top 10 countries which most frequently collaborated with other countries. Researchers from the USA had the most collaborations with other countries. Researchers from the UK, China, India and Australia frequently collaborated with those from the USA (Figure 3A). Italian researchers frequently co-worked with researchers in other areas of Europe, including Turkey, Spain, Germany and France. Researchers in Brazil, Mexico, South Korea, Portugal, Greece, Argentina, Chile, Colombia and Russia also collaborated frequently (Figure 3A).

When we used VOSviewer to conduct a cluster analysis of the co-occurrence of authors, we set the minimum number of documents for each author to five and excluded those who were not associated with the publication of other articles, resulting in the discovery of 128 authors (Figure 3B). Italian authors formed the biggest cluster. The circles of Villani, Buonsenso, and Campana were larger than those of other authors, indicating that they were the three authors who contributed the most co-authored articles.

### Cluster Analysis of Keywords

**Figure 4:**
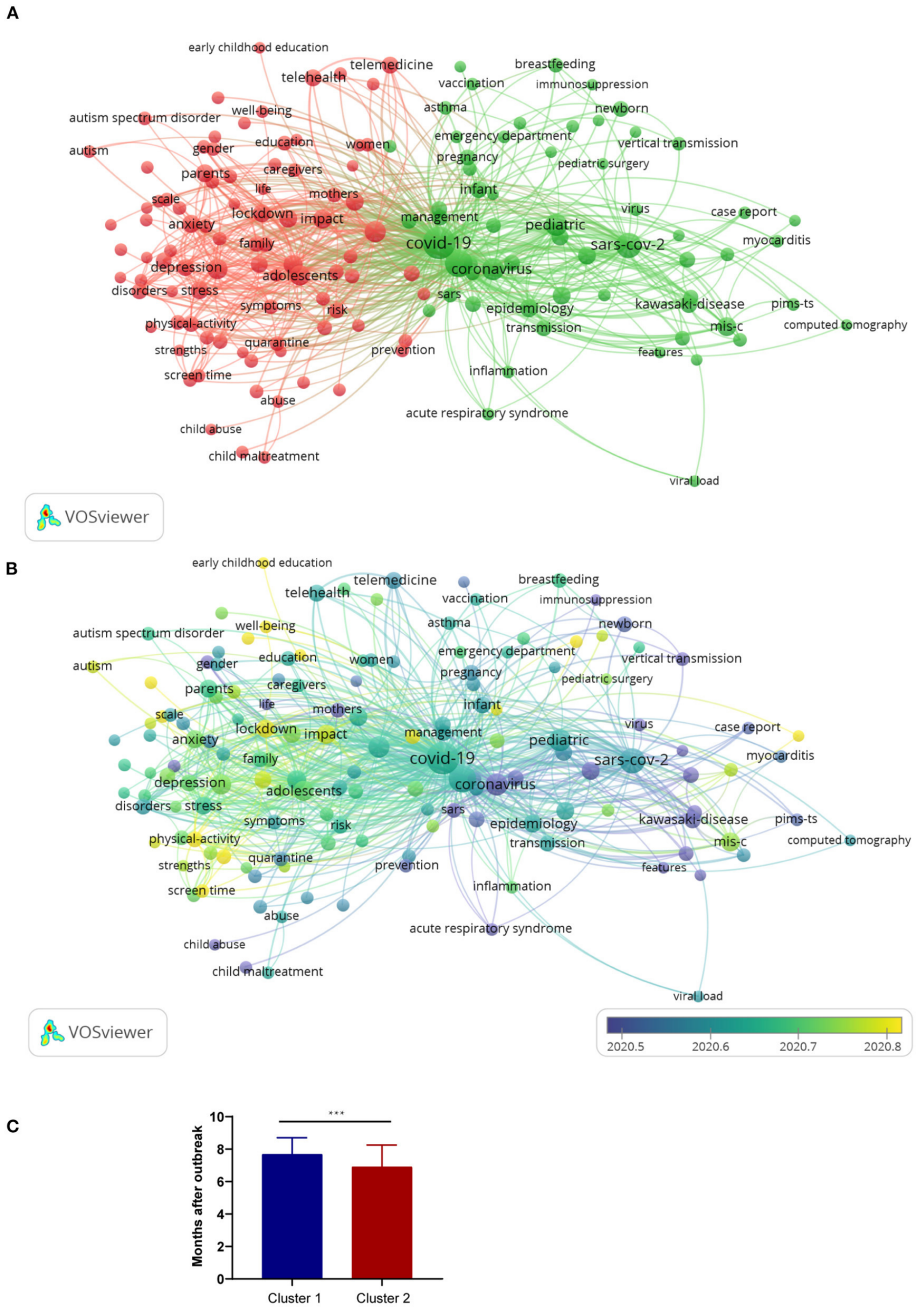
Analysis of keywords in publications on pediatric COVID-19. **(A)** A map of keywords in publications on pediatric COVID-19. The words were divided into two clusters with different colors generated by default: “social research” (cluster 1: left and red) and “clinical research” (cluster 2: right and green). A large circle indicates that the keywords appear at a high frequency. **(B)** The average frequency of appearance of the keyword is depicted from deep blue to yellow. If the keyword is closer to deep blue, then the average appearance of the keyword is higher. If the keyword is closer to yellow, then the average appearance is lesser. Two keywords were considered to co-occur if they both occurred on the same line in the main article. The smaller the distance between two keywords, the larger the number of co-occurrences of the keywords. **(C)** Comparison of clusters 1 and 2 over time: the X-axis shows the cluster, and the Y-axis shows the average month of the pandemic outbreak. ***P < 0.001.

We used VOSviewer (Center for Science and Technology Studies) to graph and analyze keywords of the 3561 articles we screened. The analytic consequence of 149 keywords with at least 15 occurrences is presented in Supplementary Table 3. The table showed the detail data including the clusters, links, occurrences, and average appearing years. After setting the minimum number of occurrences to 15 and merging synonyms, we divided the 149 keywords into two clusters: social research (cluster 1) and clinical research (cluster 2). In the social research cluster, in addition to the index words, the most frequent keywords were pandemic (280 times), adolescents (204), mental health (187 times), health (172 times), impact (148 times), care (116 times), parents (115 times), and stress (111 times). Besides the index words, the most frequent keywords in the clinical research cluster were MIS-C (144 times), infections (129 times), epidemiology (110 times), desease (104), infant (85), kawasaki-disease (83), diagnosis (76), and pneumonia (74) (Figure 4A) (Supplementary Table 3).

Colors were used to represent the appearance frequency of keywords. The closer the word was to blue, the earlier its appearance in publications, and the closer the word was to yellow, the later its appearance in publications. In the social research cluster, the earliest appearing keyword was “child protection” (cluster 1, the average appearing year (AAY) of keywords is 2020.3; 21 times), and the latest appearing keyword was “early childhood education” (cluster 1, AAY is 2020.9; 16 times). In the clinical research cluster, the earliest appearing keyword was “acute respiratory syndrome” (cluster 2, AAY is 2020.0; 24 times), and the latest appearing keyword was “serology” (cluster 2, AAY is 2020.9; 15 times) (Figure 4B) (Supplementary Table 3). We analyzed the appearing month from the keyword appearing time to the pandemic outbreak in November 2019. We found that the appearing month of cluster 1 was 7.68 ± 1.03, while the appearing month of cluster 2 was 6.91 ± 1.34. The appearing month of cluster 1 was greater than that of cluster 2 (*P* < 0.001). That means keywords in cluster 1 appeared later than keywords in cluster 2 (Figure 4C).

## Discussion

COVID-19 outbreak has resulted in global effects on adults and children. Wei et al. first published an article about COVID-19 in children in February 2020 (21). Since then, reports about this topic have significantly increased. In 2020, the number of reports was 1,357; however, by September 2021, the number had increased to 3,561. This increasing trend is consistent with the trend of the COVID-19 pandemic. More articles will be published to reveal the characteristics, effects, and treatment of pediatric COVID-19.

In another bibliometric analysis of pediatric COVID-19, their result showed that the United States, China, Italy and India were the counties that contributed the most articles by June 11, 2020. Our data showed, by September 20 2021, the United States had the most published articles, followed by Italy, the United Kingdom, and China. The published articles of United States, China and Italy maintain the increasing trends (18). The United States had the most published articles, followed by Italy, the United Kingdom, and China. China was the first country to discover and to report COVID-19. The United States experienced the highest number of new coronavirus infections and deaths. In March 2020, SARS-CoV-2 infections began to exponentially increase in the United States, placing a substantial burden on the healthcare system (22). Later, the COVID-19 pandemic forced the Italian government to enforce extreme measures and to place the entire country under lockdown conditions (23). On February 28, 2020, Lillie et al. reported the first two patients in the United Kingdom with person-to-person transmission (24). In terms of cooperation among countries, the United States cooperated most with different countries, especially Japan and India. Seven of the 10 countries that lead in cooperation are European, indicating that cooperation among European countries is quite frequent.

The 10 most highly cited articles were from the United States and the United Kingdom. Furthermore, the 10 most cited articles were related to clinical features and epidemiological characteristics of pediatric COVID-19, indicating that this is a hotspot for pediatric COVID-19 research and that researchers are focusing on this topic. The article “Epidemiology of COVID-19 Among Children in China” by Dong et al. was published during the early stages of the pandemic, enrolled 2,135 pediatric patients with COVID-19, and explored epidemiological characteristics and transmission patterns in mainland China (20). Therefore, this report has gained widespread attention and has been widely cited.

Among the 10 institutions with the most published articles, 70% were in the United States. Among the 10 institutions with the most frequently cited articles, 40% were in the United States, indicating that the United States performs more research on this topic. It is worth noting that of the 10 institutions with the most publications, only one was in China; however, five of the 10 institutions with the most frequently cited articles were in China. Huazhong University of Science and Technology published the most articles and the citation numbers was the top two institutions. Shanghai Jiao Tong University, with 16 articles, is not among the 10 institutions with the most articles; however, it has the most frequently cited articles. Xi'an Jiao Tong University, Nanjing Medical University and Anhui Medical University published <10 articles but their article owned high citation. The reason may due to that China was the first country to study and report cases of COVID-19, and its effective treatment experience has provided a clinical reference for more physicians and scholars. The University of Pennsylvania was the institution with the third highest number of published articles and these articles were the most frequently cited in the institutional analysis.

Compared with Monzani et al.'s article (18), the most highly cited articles and top 10 institutions were different. The reason is not just due to the time of bibliometric analysis, but because of the different search strategies. To focus on the specific pediatric trends, the search strategy that we used was keywords in author keywords and titles. To cover all the children, besides pediatric and child, we also used the keywords including baby, newborn, infant, and toddler.

The authors Alberto Villani from the Bambino Gesù Children's Hospital, IRCCS, in Italy and Shao Jianbo from Huazhong University of Science and Technology in China have published the most articles about COVID-19 in children and have performed more research in the field and might be more knowledgeable about research trends. Therefore, following their research developments and trends may help us understand the forefront of pediatric COVID-19 academics. For example, researchers focused on clinically relevant research of COVID-19 in children, such as the diagnosis (25, 26), clinical features (27, 28), clinical management (29–31), and treatment of COVID-19 in combination with other diseases (32, 33). The articles by Shao are mainly related to the differentiation of COVID-19 and other pulmonary infections in children (34–36), clinical and computed tomography features (37–40), and COVID-19-related immunology studies (41–43).

*Pediatrics* was one of the most authoritative journals and was classified as Q1 by JCR. The impact factor of *Pediatrics* was the highest of the top 10 most active journals. The aforementioned most cited article was published by *Pediatrics* (20). The articles from *Pediatrics* were widely cited by researchers. *Frontiers in Pediatrics* contributed the most pediatric COVID-19 articles, but was not the most cited journal. The *Pediatric Infectious Disease Journal* was the journal which published the second largest number of articles and had the second largest number of citations, after *Pediatrics*. We can, therefore, follow these active journals to learn the trends of pediatric COVID-19.

Researchers in the USA collaborated with the most countries, including the UK, China, India and Australia. Together, these countries formed the biggest cluster of collaborating countries. Researchers in European countries frequently cooperated with each other and formed the second cluster of collaborating countries. Italian researchers most frequently worked with other European researchers, which may due to the geographical position and the widespread incidence of COVID-19 in Italy (44). Of the collaborating authors, the biggest cluster was from Italy.

In terms of co-authorship, Alberto Villani, Danilo Buonsenso and Andrea Campana, who are all from Rome, Italy, had the largest number of collaborations. Alberto Villani and Andrea Campana are both affiliated with the University Department of Pediatrics, Bambino Gesù Children's Hospital. Danilo Buonsenso is affiliated with the Department of Woman and Child Health and Public Health, Fondazione Policlinico Universitario A. These authors are all in the rank of top 10 greatest number of publications. They have contributed the most co-authored articles and have close academic ties with other authors, which allows us to more clearly recognize this excellent team of scholars.

Our analysis of the clinical research related keywords identified that the most frequent keywords were MIS-C, infections, epidemiology, disease, infant, Kawasaki-disease, diagnosis, and pneumonia. These words draw more attention to COVID-19 research. Initially, the clinical manifestations of pediatric COVID-19 were unknown; therefore, manifestation and diagnosis were the most important keywords. The morbidity and mortality of COVID-19 disease in children were low, but MIS-C and Kawasaki disease are more serious diseases which occur as a result of SARS-CoV-2 infection (7, 8). Kawasaki-like disease associated with COVID-19 has been increasingly reported. Large case series of Kawasaki disease related to SARS-CoV-2 infection from the UK, Italy, America, and France have been published (45). MIS-C is a newly defined condition, caused by a storm of inflammatory factors associated with COVID-19. Critically ill children with COVID-19 associated MIS-C have a spectrum of severity broad damage (46). Similar to Kawasaki disease, MIS-C has multi-system involvement and can lead to severe myocardial damage (47).

Our results showed that pandemic, adolescents, mental health, health, impact, care, parents, and stress were the frequently occurrence keywords. These keywords appeared later than the clinical research. This remind us the new hotspot for pediatric COVID-19 research is gradually shifting from COVID-19 itself and its related clinical studies to studies of the psychological impact and social impact on children; this may be because, as the epidemic continues, home isolation, social distancing, and fear of disease have had significant effects on the schooling, lives, and mental health of children (16, 48). The World Health Organization focusses their attention on the social effects of COVID-19 on children and adolescents, including ensuring safe schooling, minimizing the disruption of health systems for children, safeguarding the quality of their care and so on (49).

The data analysis is comprehensive and objective. However, some limitations remain. Firstly, we only enrolled publications in English which may exclude some important non-English studies related to the pediatric COVID-19. Second, because we need keywords to analyze the research trends, we excluded the letters in bibliometric analysis. Moreover, as the database is still being updated, the bibliometric analysis results may differ slightly from the present research situation.

In conclusion, this bibliometric analysis provided an overview of pediatric COVID-19 studies and identified some significant issues. The topic of pediatric COVID-19 has attracted considerable attention worldwide, thus leading to a considerable number of articles published over the past 2 years. This study demonstrated that the United States, China, and Italy have leading roles in pediatric COVID-19 research. Furthermore, the new research hotspot is gradually shifting from COVID-19 and related clinical studies to studies of psychological and social impacts on children.

## Data Availability Statement

The raw data supporting the conclusions of this article will be made available by the authors, without undue reservation.

## Author Contributions

SH and HC conceived the study. SH, YM, and XW analyzed the data. HC designed and supervised the study. All authors contributed to the data analysis and reviewed and edited the manuscript.

## Funding

This work was supported by the National Natural Science Foundation of China (Grant No. 81800021) and the Jilin Provincial Key Laboratory of Biotherapy (Grant No. 20200201464JC).

## Conflict of Interest

The authors declare that the research was conducted in the absence of any commercial or financial relationships that could be construed as a potential conflict of interest.

## Publisher's Note

All claims expressed in this article are solely those of the authors and do not necessarily represent those of their affiliated organizations, or those of the publisher, the editors and the reviewers. Any product that may be evaluated in this article, or claim that may be made by its manufacturer, is not guaranteed or endorsed by the publisher.
