# Supplementary material for: Global Research Trends in Pediatric COVID-19: A Bibliometric Analysis
Source: Front Public Health. 2022 Feb 16;10:798005. doi: 10.3389/fpubh.2022.798005 (PMC8888448; doi:10.3389/fpubh.2022.798005)
Supplement: Supplementary file 1 [file Table_1.docx]

**Supplementary Table 1. Summary of the publications**

| Item | Number |
| --- | --- |
| Total retrieved publications | 6510 |
| Total selected articles | 3563 |
| Articles in 2020 | 1359 |
| Articles in 2021 by September 20 | 2204 |
| Countries | 138 |
| Journals | 1139 |
