# Supplementary material for: Global Research Trends in Pediatric COVID-19: A Bibliometric Analysis
Source: Front Public Health. 2022 Feb 16;10:798005. doi: 10.3389/fpubh.2022.798005 (PMC8888448; doi:10.3389/fpubh.2022.798005)
Supplement: Supplementary file 2 [file Table_2.docx]

**Supplementary Table 2 Information of co-occurrences countries**

| No. | Country | Cluster | Links | Total link strength |
| --- | --- | --- | --- | --- |
| 1 | USA | 2 | 39 | 652 |
| 2 | UK | 2 | 39 | 590 |
| 3 | Italy | 1 | 37 | 412 |
| 4 | Spain | 1 | 37 | 351 |
| 5 | Germany | 1 | 37 | 281 |
| 6 | France | 1 | 37 | 276 |
| 7 | Australia | 2 | 35 | 244 |
| 8 | Switzerland | 1 | 34 | 242 |
| 9 | Canada | 5 | 35 | 226 |
| 10 | Netherlands | 1 | 37 | 220 |
| 11 | China | 2 | 38 | 210 |
| 12 | India | 2 | 37 | 186 |
| 13 | Belgium | 1 | 32 | 181 |
| 14 | Brazil | 3 | 36 | 177 |
| 15 | Poland | 1 | 35 | 166 |
| 16 | Sweden | 1 | 36 | 160 |
| 17 | Turkey | 1 | 32 | 118 |
| 18 | Austria | 1 | 29 | 117 |
| 19 | Ireland | 1 | 29 | 117 |
| 20 | Egypt | 4 | 31 | 101 |
| 21 | Mexico | 3 | 29 | 93 |
| 22 | Japan | 2 | 30 | 91 |
| 23 | Greece | 3 | 30 | 90 |
| 24 | Colombia | 3 | 29 | 89 |
| 25 | Denmark | 1 | 29 | 86 |
| 26 | South Africa | 2 | 27 | 85 |
| 27 | Argentina | 3 | 31 | 82 |
| 28 | Israel | 5 | 25 | 81 |
| 29 | Pakistan | 2 | 29 | 75 |
| 30 | Russia | 3 | 30 | 74 |
| 31 | Singapore | 2 | 27 | 71 |
| 32 | Chile | 3 | 25 | 69 |
| 33 | Saudi Arabia | 4 | 26 | 66 |
| 34 | Portugal | 3 | 24 | 64 |
| 35 | U Arab Emirates | 4 | 22 | 57 |
| 36 | Finland | 1 | 25 | 46 |
| 37 | South Korea | 3 | 21 | 42 |
| 38 | Iran | 2 | 19 | 40 |
| 39 | Indonesia | 2 | 15 | 34 |
| 40 | Nigeria | 2 | 13 | 30 |
