# Supplementary material for: Global Research Trends in Pediatric COVID-19: A Bibliometric Analysis
Source: Front Public Health. 2022 Feb 16;10:798005. doi: 10.3389/fpubh.2022.798005 (PMC8888448; doi:10.3389/fpubh.2022.798005)
Supplement: Supplementary file 3 [file Table_3.docx]

**Supplementary Table 3 Information of occurrences keywords**

| No. | Keyword | cluster | Links | Occurrences | Average appearing years（AAY) |
| --- | --- | --- | --- | --- | --- |
| 1 | covid-19 | 2 | 148 | 2316 | 2020.645 |
| 2 | children | 2 | 143 | 1016 | 2020.63 |
| 3 | sars-cov-2 | 2 | 114 | 685 | 2020.595 |
| 4 | pediatric | 2 | 110 | 366 | 2020.621 |
| 5 | coronavirus | 2 | 132 | 317 | 2020.486 |
| 6 | pandemic | 1 | 131 | 280 | 2020.642 |
| 7 | adolescents | 1 | 121 | 204 | 2020.708 |
| 8 | mental-health | 1 | 110 | 187 | 2020.726 |
| 9 | health | 1 | 119 | 172 | 2020.667 |
| 10 | impact | 1 | 115 | 148 | 2020.772 |
| 11 | mis-c | 2 | 50 | 144 | 2020.74 |
| 12 | infections | 2 | 87 | 129 | 2020.5 |
| 13 | care | 1 | 100 | 116 | 2020.647 |
| 14 | parents | 1 | 95 | 115 | 2020.674 |
| 15 | stress | 1 | 90 | 111 | 2020.663 |
| 16 | covid-19 pandemic | 1 | 84 | 110 | 2020.729 |
| 17 | epidemiology | 2 | 88 | 110 | 2020.615 |
| 18 | anxiety | 1 | 93 | 106 | 2020.721 |
| 19 | lockdown | 1 | 92 | 105 | 2020.798 |
| 20 | disease | 2 | 90 | 104 | 2020.62 |
| 21 | telemedicine | 1 | 65 | 100 | 2020.577 |
| 22 | depression | 1 | 97 | 96 | 2020.709 |
| 23 | telehealth | 1 | 67 | 89 | 2020.615 |
| 24 | infant | 2 | 83 | 85 | 2020.6 |
| 25 | kawasaki-disease | 2 | 42 | 83 | 2020.507 |
| 26 | diagnosis | 2 | 82 | 76 | 2020.575 |
| 27 | prevalence | 1 | 97 | 76 | 2020.769 |
| 28 | family | 1 | 80 | 75 | 2020.683 |
| 29 | pneumonia | 2 | 54 | 74 | 2020.405 |
| 30 | pregnancy | 2 | 72 | 73 | 2020.574 |
| 31 | physical-activity | 1 | 70 | 70 | 2020.8 |
| 32 | outbreak | 2 | 86 | 69 | 2020.523 |
| 33 | outcome | 2 | 83 | 69 | 2020.614 |
| 34 | risk | 1 | 93 | 69 | 2020.645 |
| 35 | united-states | 1 | 74 | 66 | 2020.712 |
| 36 | behaviors | 1 | 81 | 63 | 2020.727 |
| 37 | disorders | 1 | 78 | 61 | 2020.6 |
| 38 | management | 2 | 76 | 61 | 2020.62 |
| 39 | transmission | 2 | 64 | 61 | 2020.617 |
| 40 | china | 2 | 66 | 55 | 2020.51 |
| 41 | newborn | 2 | 42 | 55 | 2020.519 |
| 42 | education | 1 | 69 | 53 | 2020.63 |
| 43 | quarantine | 1 | 85 | 53 | 2020.563 |
| 44 | mothers | 1 | 90 | 52 | 2020.476 |
| 45 | symptoms | 1 | 87 | 52 | 2020.605 |
| 46 | associations | 1 | 90 | 50 | 2020.696 |
| 47 | school | 1 | 77 | 50 | 2020.625 |
| 48 | mortality | 2 | 58 | 48 | 2020.732 |
| 49 | youth | 1 | 72 | 48 | 2020.711 |
| 50 | time | 1 | 78 | 46 | 2020.684 |
| 51 | clinical characteristics | 2 | 33 | 44 | 2020.488 |
| 52 | obesity | 1 | 62 | 44 | 2020.829 |
| 53 | public health | 2 | 58 | 43 | 2020.821 |
| 54 | childhood | 1 | 72 | 42 | 2020.714 |
| 55 | sars | 2 | 76 | 42 | 2020.3 |
| 56 | breastfeeding | 2 | 36 | 41 | 2020.676 |
| 57 | wuhan | 2 | 34 | 41 | 2020.275 |
| 58 | covid | 2 | 54 | 40 | 2020.595 |
| 59 | women | 1 | 64 | 38 | 2020.611 |
| 60 | experiences | 1 | 70 | 37 | 2020.607 |
| 61 | parenting | 1 | 64 | 37 | 2020.615 |
| 62 | influenza | 2 | 55 | 36 | 2020.546 |
| 63 | multisystem inflammatory syndrome | 2 | 33 | 36 | 2020.758 |
| 64 | prevention | 1 | 51 | 36 | 2020.516 |
| 65 | sleep | 1 | 70 | 36 | 2020.742 |
| 66 | resilience | 1 | 71 | 35 | 2020.567 |
| 67 | screen time | 1 | 56 | 33 | 2020.862 |
| 68 | shock | 2 | 28 | 33 | 2020.581 |
| 69 | epidemic | 1 | 65 | 32 | 2020.586 |
| 70 | quality of life | 1 | 60 | 32 | 2020.72 |
| 71 | school closure | 1 | 53 | 31 | 2020.692 |
| 72 | vertical transmission | 2 | 25 | 31 | 2020.37 |
| 73 | autism spectrum disorder | 1 | 46 | 30 | 2020.667 |
| 74 | coronavirus infections | 2 | 35 | 30 | 2020.586 |
| 75 | scale | 1 | 62 | 30 | 2020.6 |
| 76 | asthma | 2 | 42 | 29 | 2020.63 |
| 77 | intervention | 1 | 62 | 29 | 2020.583 |
| 78 | sedentary behavior | 1 | 40 | 29 | 2020.714 |
| 79 | caregivers | 1 | 64 | 28 | 2020.652 |
| 80 | gender | 1 | 51 | 28 | 2020.609 |
| 81 | trauma | 1 | 53 | 28 | 2020.577 |
| 82 | communication | 1 | 63 | 27 | 2020.5 |
| 83 | psychological impact | 1 | 63 | 27 | 2020.565 |
| 84 | quality | 1 | 54 | 27 | 2020.75 |
| 85 | abuse | 1 | 53 | 26 | 2020.619 |
| 86 | case report | 2 | 24 | 26 | 2020.52 |
| 87 | child maltreatment | 1 | 29 | 26 | 2020.632 |
| 88 | vaccination | 2 | 36 | 26 | 2020.636 |
| 89 | validity | 1 | 62 | 26 | 2020.684 |
| 90 | pims-ts | 2 | 24 | 25 | 2020.524 |
| 91 | acute respiratory syndrome | 2 | 37 | 24 | 2020.208 |
| 92 | autism | 1 | 40 | 24 | 2020.773 |
| 93 | myocarditis | 2 | 22 | 24 | 2020.546 |
| 94 | risk-factors | 1 | 57 | 24 | 2020.682 |
| 95 | type 1 diabetes | 1 | 31 | 24 | 2020.727 |
| 96 | guidelines | 1 | 55 | 23 | 2020.619 |
| 97 | sars-cov-2 infection | 2 | 27 | 23 | 2020.727 |
| 98 | social support | 1 | 50 | 23 | 2020.905 |
| 99 | survey | 1 | 38 | 23 | 2020.696 |
| 100 | technology | 1 | 46 | 23 | 2020.591 |
| 101 | well-being | 1 | 41 | 23 | 2020.8 |
| 102 | childcare | 1 | 24 | 22 | 2020.727 |
| 103 | child protection | 1 | 20 | 21 | 2020.333 |
| 104 | maltreatment | 1 | 41 | 21 | 2020.579 |
| 105 | virus | 2 | 36 | 21 | 2020.429 |
| 106 | adults | 1 | 47 | 20 | 2020.647 |
| 107 | emergency department | 2 | 28 | 20 | 2020.647 |
| 108 | inflammation | 2 | 37 | 20 | 2020.706 |
| 109 | parenting stress | 1 | 54 | 20 | 2020.625 |
| 110 | participation | 1 | 38 | 20 | 2020.8 |
| 111 | responses | 2 | 49 | 20 | 2020.737 |
| 112 | validation | 1 | 61 | 20 | 2020.722 |
| 113 | adhd | 1 | 41 | 19 | 2020.867 |
| 114 | adjustment | 1 | 45 | 19 | 2020.667 |
| 115 | age | 1 | 52 | 19 | 2020.563 |
| 116 | cancer | 2 | 34 | 19 | 2020.692 |
| 117 | child health | 2 | 24 | 19 | 2020.588 |
| 118 | questionnaire | 1 | 53 | 19 | 2020.462 |
| 119 | infectious disease | 2 | 24 | 18 | 2020.688 |
| 120 | social distancing | 1 | 31 | 18 | 2020.571 |
| 121 | vaccine | 2 | 29 | 18 | 2020.833 |
| 122 | ace2 | 2 | 31 | 17 | 2020.412 |
| 123 | burden | 2 | 36 | 17 | 2020.786 |
| 124 | congenital heart disease | 2 | 21 | 17 | 2020.765 |
| 125 | early childhood | 1 | 23 | 17 | 2020.769 |
| 126 | emergency | 2 | 34 | 17 | 2020.6 |
| 127 | health-care | 1 | 44 | 17 | 2020.533 |
| 128 | pediatric surgery | 2 | 22 | 17 | 2020.733 |
| 129 | reliability | 1 | 49 | 17 | 2020.692 |
| 130 | surgery | 2 | 20 | 17 | 2020.533 |
| 131 | childbirth | 1 | 33 | 16 | 2020.818 |
| 132 | computed tomography | 2 | 13 | 16 | 2020.6 |
| 133 | confinement | 1 | 43 | 16 | 2020.714 |
| 134 | early childhood education | 1 | 17 | 16 | 2020.933 |
| 135 | fear | 1 | 43 | 16 | 2020.643 |
| 136 | features | 2 | 26 | 16 | 2020.5 |
| 137 | food insecurity | 1 | 36 | 16 | 2020.75 |
| 138 | strengths | 1 | 51 | 16 | 2020.75 |
| 139 | antibodies | 2 | 26 | 15 | 2020.667 |
| 140 | child abuse | 1 | 27 | 15 | 2020.455 |
| 141 | depressive symptoms | 1 | 45 | 15 | 2020.6 |
| 142 | immunosuppression | 2 | 19 | 15 | 2020.462 |
| 143 | kawasaki-like disease | 2 | 23 | 15 | 2020.5 |
| 144 | life | 1 | 50 | 15 | 2020.5 |
| 145 | metaanalysis | 1 | 43 | 15 | 2020.714 |
| 146 | serology | 2 | 15 | 15 | 2020.933 |
| 147 | social media | 1 | 39 | 15 | 2020.667 |
| 148 | students | 1 | 37 | 15 | 2020.778 |
| 149 | viral load | 2 | 11 | 15 | 2020.6 |
